# FAIR digital objects in environmental and life sciences should comprise workflow operation design data and method information for repeatability of study setups and reproducibility of results

**DOI:** 10.1093/database/baaa059

**Published:** 2020-08-20

**Authors:** Janno Harjes, Anton Link, Tanja Weibulat, Dagmar Triebel, Gerhard Rambold

**Affiliations:** 1 University of Bayreuth, Universitätsstraße 30, 95440 Bayreuth, Germany; 2 Staatliche Naturwissenschaftliche Sammlungen Bayerns, Menzinger Straße 67, 80638 München, Germany; 3 German Federation for Biological Data e. V., Campus Ring 1, 28759 Bremen, Germany

## Abstract

Repeatability of study setups and reproducibility of research results by underlying data are major requirements in science. Until now, abstract models for describing the structural logic of studies in environmental sciences are lacking and tools for data management are insufficient. Mandatory for repeatability and reproducibility is the use of sophisticated data management solutions going beyond data file sharing. Particularly, it implies maintenance of coherent data along workflows. Design data concern elements from elementary domains of operations being transformation, measurement and transaction. Operation design elements and method information are specified for each consecutive workflow segment from field to laboratory campaigns. The strict linkage of operation design element values, operation values and objects is essential. For enabling coherence of corresponding objects along consecutive workflow segments, the assignment of unique identifiers and the specification of their relations are mandatory. The abstract model presented here addresses these aspects, and the software *DiversityDescriptions (DWB-DD)* facilitates the management of thusly connected digital data objects and structures. *DWB-DD* allows for an individual specification of operation design elements and their linking to objects. Two workflow design use cases, one for DNA barcoding and another for cultivation of fungal isolates, are given. To publish those structured data, standard schema mapping and XML-provision of digital objects are essential. Schemas useful for this mapping include the Ecological Markup Language, the Schema for Meta-omics Data of Collection Objects and the Standard for Structured Descriptive Data. Data pipelines with *DWB-DD* include the mapping and conversion between schemas and functions for data publishing and archiving according to the Open Archival Information System standard. The setting allows for repeatability of study setups, reproducibility of study results and for supporting work groups to structure and maintain their data from the beginning of a study. The theory of ‘FAIR++’ digital objects is introduced.

## Introduction

A ‘replication crisis’ and ‘reproducibility crisis’ in natural sciences have been under intensified discussion since recently (1, 2, 3) and address the paradigm that scientists should be enabled to better repeat study setups and reproduce study results in the future. Particularly in life sciences including ecology, there is, for several reasons, a lack of empirical studies, which tested earlier research findings by repetition (4). Reasons for the actual crisis are manifold (5). Regarding ecological and evolution research, they have been exemplarily analysed (6). The challenge is connected with the task to produce, document and report on all domains and all kind of data assets generated during the research process. Incomplete and wrong data might only rarely have been generated by intention, but unintentionally without having been recognized as such (7).

In environmental sciences, including ecology, the generation of flawed data may occur already in the field due to confusion of objects or object containers, at subsequent stages, due to mislabelling or to errors during laboratory operations (8). In collaborative biodiversity studies describing and analysing species community structure and molecular, cellular and organismic interactions such errors may be particularly frequent due to shortcomings during early phases of data management (9). Flat-structured data editing tools like spreadsheets have often been recognized as sufficient, probably due to the fact that data management during an early project phase is considered being less relevant and being under technicians’ stewardship. Certainly, for estimating quality and reliability of data products to be analysed, it is mandatory that all research process participants are involved to a considerable degree in the early data management. First practical guidelines to cope with this issue, particularly in long-term ecological monitoring projects, are available (10). Other researchers point to the lack of adequate basic data management procedures and the lack of infrastructure and of significant human resources (11).

Recently, research data management in context with data publication following FAIR data principles has become a major topic and has been addressed by international and national initiatives (e.g. FAIRsharing, GFBio project in Germany) (12, 13, 14, 15, 16). The demand of FAIRness has also strengthened evaluation and certification activities in the landscape of recognized scientific data repositories at various organization levels regarding transparency, interoperability and reusability of data for avoiding the creation of ‘data silos’ (17, 18).

Compared to requirements of data reusability (19), requirements of ‘repeatability’ of study setups and ‘reproducibility’ of research results go one step further, meeting study operation design, methods applied, data provenience and dataflow details (20, 21). Reusability may often be considered being a problem of the users, i.e. data consumers, how to handle, i.e. further process the accessible identified data products, i.e. data sets, for their own use (09) and may also be regarded as a problem of appropriate data preservation (sometimes together with software applied) and of the assignment of relevant properties and ontologies (22, 23). Regarding repeatability of study setups and reproducibility of resulting data, available data products often appear to be insufficient in completeness, quality and extent of documentation of relations between operation design and method information.

Digital objects are generated along all steps of the workflow in a scientific study. Coming from object-oriented programming, the term has been defined as ‘a unit of information that includes properties (attributes or characteristics of the object) and may also include methods (means of performing operations on the object)’, see https://www2.archivists.org/glossary/terms/d/digital-object. Used in a more general context, digital objects are meaningful entities in the digital domain having names (identities) and properties as well (24). The Digital Object Architecture addressing interoperability in heterogeneous networks, defines the term ‘digital object (DO)’ as ‘a sequence of bits, or a set of sequences of bits, incorporating a work or portion of a work or other information in which a party has rights or interests, or in which there is value, each of the sequences being structured in a way that is interpretable by one or more of the computational facilities, and having as an essential element an associated unique persistent identifier’ (25). This definition has recently been reflected and accepted for data and services in biodiversity science and geoscience (26) and is followed here.

The digital objects generated in a research study often insufficiently reflect the provenience and relations of objects, meaning vertical, i.e. synchronous, and horizontal, i.e. successional data **coherence** or **concatenation**, respectively, as well as applied information structures, formats, standard schemas and ontologies. Thus, the study workflow with its segments and results as a whole is not representable. Within the last years, the workflow for publication of scientific result data has been improved (27). Still insufficient attention, however, has been given to data management during early processes for generating data products as one form of digital objects and documentation of data handling, which is a prerequisite for reusable and reproducible scientific results. This frequently resulted in data products with structured or semi-structured non-standardized content in various technical formats, along with certain standardized bibliographic information only (28), deposited in non-domain-specific ‘file sharing’ data repositories (09).

The present contribution describes an abstract model. It is based on three elementary operation domains for all segments along research workflows to obtain highly structured data products. Such granular modelling approach is preconditional for generating interoperable bioscience and environmental data (29). The model describes scientific workflows as concatenated segments. Generated data products or, more general, generated digital objects comprise all information for the documentation of a study. This information should guarantee the repeatability of the conditions for observation and thus might allow—if all influential factors could be kept under control—for reproducibility of study results. This article supplements two preceding ones, which are also dedicated to the management of environmental research data (30, 31).

## Challenges of scientific data management

Environmental research focuses on the complexity of interactions in nature. A variety of observational and experimental setups are required for testing evolving scientific questions or hypotheses. Thus, specific challenges of scientific data management exist. To cope with these, the setup of appropriate data management plans (DMPs) is regarded as mandatory. Such DMPs should provide study design information and concepts of how to achieve reproducibility and ‘FAIRness’ of resulting data, as well as repeatability of experiments (19, 32, 33). Furthermore, electronic laboratory notebooks (ELNs) or journals are used for documenting analysis data gained during operations in the laboratory. In addition, methodologies and scientific workflows applied during a study are documented in text documents and more recently, in Scientific Data Management Systems (SDMS), often being an integral part of laboratory information managment systems (LIMS). Finally, parts of the information on applied methods are described and referred to in the methodological chapters of resulting original publications. There is also increasing awareness that physical objects (environmental and other samples) require deposition in relevant material repositories (34, 35). Digital objects with data from measurements (along with design data and methodology information) from scientific workflows are supposed to be stored in relevant institutional or domain-specific, regional, national or international data repositories (e.g. in those recommended by journals, by national funding agencies or by data infrastructure consortia like the German Federation for Biological Data (GFBio) (36).

Community-agreed conceptual schemas for describing discipline-specific operations and measurement data provide a more or less comprehensive namespace for ‘variables’ and ‘parameters’, being utilized as elements or descriptors in data management systems, data exchange documents and online data portals. However, it is another challenge to implement such schemas in standard database applications or virtual research environments because they may either be too generic, patchy or too specific to be used in scientific studies with different experimental setups. This entails that on the one hand, study designs should be specific enough according to the scientific questions or hypotheses by use of discipline-specific ontologies, and on the other, descriptors should be suitable to be translated onto namespace elements and ontologies of community-agreed schemas.

The following abstract model addresses some of these challenges. Its applicability and suitability in practice has been tested by real-world use cases from ecological field and laboratory studies using an established generic SDMS.

## Abstract model for analysing and describing FAIR digital objects in environmental and life sciences, and steps towards practice

The achieved characteristics of the new model include the abstraction of workflow segmentation, workflow segment design, design elements and method information, the generation of measurement values, object identity and object identifiers and the linking of workflow segments, operation designs and methods with design codes. Two use cases from environmental and life sciences are added. Details on related software implementation and considerations on mapping to standard conceptual schemas and ontologies are provided.

### Workflow segmentation

During workflows in environmental research, a given number of physical objects and corresponding digital data objects are generated, the first by transformation of a preceding physical object, the latter by measurements on the physical object in focus or by transformation of a preceding digital data object. Therefore, a workflow from environmental sampling to data analysis in the laboratory is potentially divisible into a series of **workflow segments** according to the respective number of generated (intermediate or final) physical (or digital) objects. This means, in a most narrow concept, a workflow segment may be demarcated by only one object and corresponding measurement data (31). In a study, one to several workflow segments may constitute a study **campaign**. The combination of more than one segment in a campaign is due to practical reasons. The linkages between the segments within campaigns or along a whole workflow can be achieved by applying physical (or digital) object identifier relations, mainly parent identity relation. Object identifiers are identifiers used for defining the physical and digital object or unit identity (30). Further explanations are given in the chapter ‘Object Identity and Object Identifiers’ below and in Figure 1.

**Figure 1:**
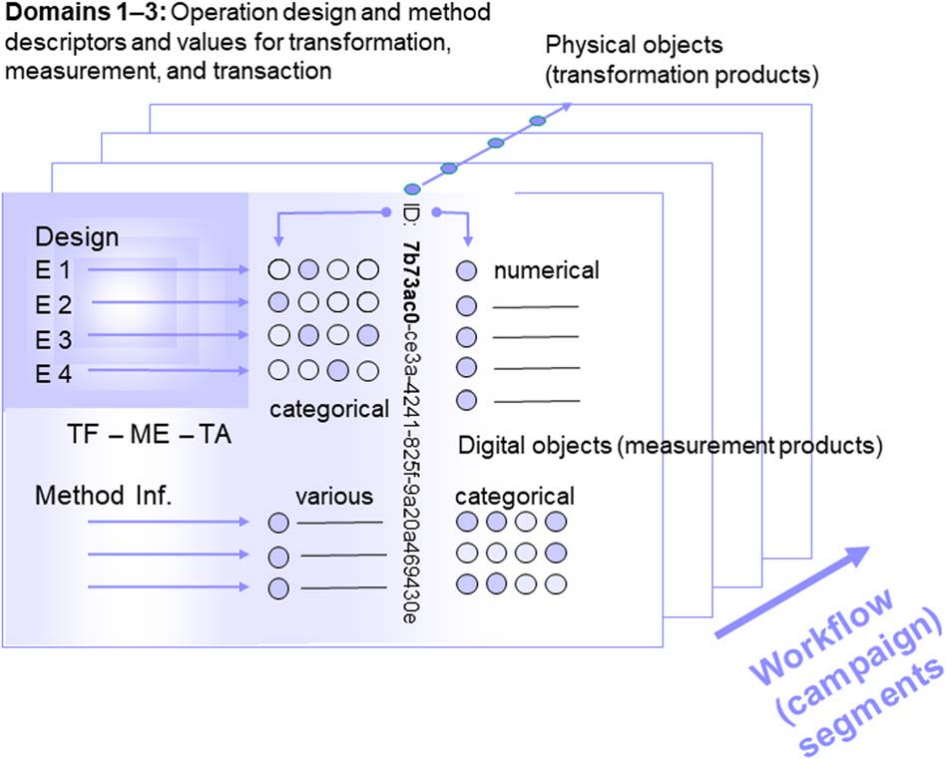
Segment of a (multi-segment) workflow or a (multi-segment) campaign with object identity (ID), operation design elements and method information, as well as measurement values as assigned to physical (and digital) objects. Consecutive segments (indicated by the arrow at the lower right) are linked via parent identifier relations of the preceding physical objects or their digital representatives (parent identity relation). Operations are grouped according to the domains transformation, measurement and transaction (TF: transformation design, referring to domain 1, ME: method design, referring to domain 2, TA: transaction design, referring to domain 3), being assigned to (physical and digital) objects by declaration or selection of descriptor states (categorical, various). Measurement (or observation) values are primarily generated from physical objects (and secondarily from digital objects).

### Workflow segment design and object description

Workflow segments are composed of physical or digital **objects**, which are characterized by operation data and method information (Figure 1) according to a given **study design**. Operations within the sequential workflow steps concern activities in the field and in the laboratory including object storage. In environmental research, particularly design data of field activities, like sampling and measurement of spatiotemporal coordinates, are required for interpreting data gained later in the workflow by measurements taken from physical objects during subsequent workflow segments. This means that measurement design-based data are required for correlation with transformation design-based data, to test scientific hypotheses as well as for quality and quantity control of a given object in a workflow segment. Complete sets of data describing study and workflow (segment) designs are essential for the repeatability of study setups and the potential reproducibility of results. This includes all types of research projects and studies with theory-driven and data-driven study design and research perspectives (37).

**Design elements** describing the **object contextual properties** as well as **method information** may be assigned to domains according to the three **elementary operations**. The **‘transformation design (element)’** (TF) refers to **domain 1**, **‘measurement design’** (ME) to **domain 2** and **‘transaction design’** (TA) to **domain 3**. While domains 1 to 3 design elements specify e.g. spatial or other hierarchies in assays for transformations, storage, measurements and transfer, method information describes devices as well as the details and parameters for operations on (physical, digital) objects. **Transformations** based on the transformation design concern every kind of invasive treatment of an object; it may also include the storage of objects. **Digital objects** generated by **measurement,** based on the measurement design, concern all kind of data that are (non-invasively) generated from a physical and (secondarily) from a digital object, describing object traits and representing the **description of objects,** which have been generated by the transformation of a preceding object according to a given transformation design and method information. **Transactions** based on the transaction design concern object transfer (i.e. translocation or transport). The elements of all three design domains together represent the workflow segment designs, the overall study design and designs of individual digital objects (31).

### Operation design elements and method information

An overall study design or the designs per workflow segment and assigned physical resp. digital objects usually include design elements of three domains (Figure 2) according to the elementary operations (31). **Designs**, which are composed by **design elements** (Table 1), represent the **variables** in a study and must be defined before starting a campaign. During data analysis after the campaigns focusing on physical objects, they represent the **factors** that are used for creating results to characterize the environmental conditions of an object. **Transformation designs** (TF) (domain 1) often follow nested or crossed designs (38). Data objects gained by measurements taken from a physical (or digital) object for quality control or for gathering trait information follow the **measurement design** (ME) (domain 2). The **transaction design** (TA) (domain 3) also mainly reflects the environmental conditions and spatial position of objects in the context of storage (room, freezer, shelf, microplate, etc.) and transport. The designs of material repositories and data repositories are usually hierarchical or nested like those for transformation. Furthermore, storage may also be regarded as a kind of transformation (e.g. due to physical or chemical changes over time) and therefore follow a transformation design accordingly.

**Figure 2:**
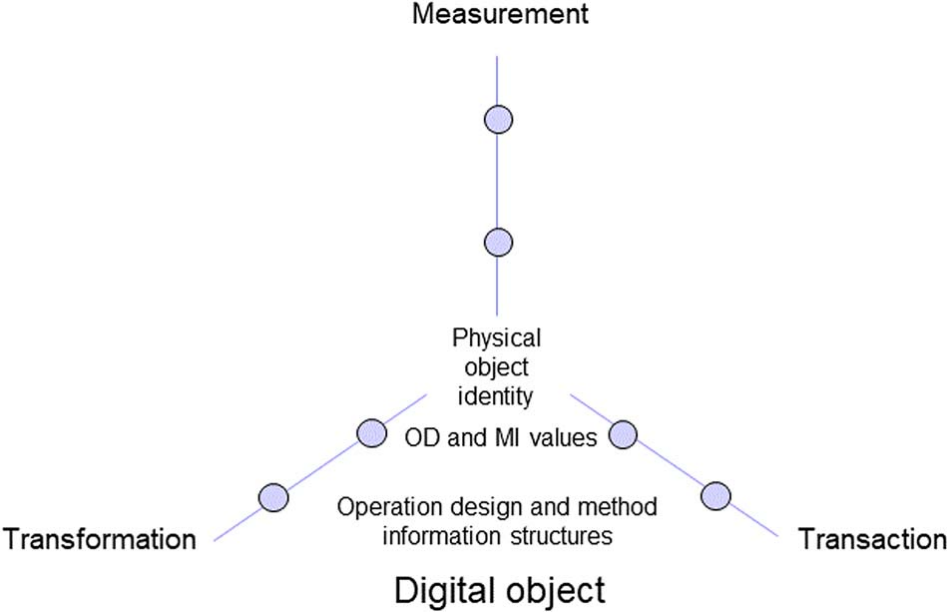
Digital object including information on physical object identity as well as operation design (OD) and method information (MI) structures and values of three elementary operation domains.

**Table 1:** Definitions of core terms

**Workflow and dataflow** Workflow → A sequence of a given number of generated physical objects and corresponding digital data objects Workflow segment → Section of workflow, defined by a given object, the design of its generation, and its properties or traits; pre- (and post-)campaign activities are not considered as part of a workflow segment Study or workflow campaign → Section of workflow, defined by the practical work in a study, given number of workflow segments (1–n) according to the number of generated (physical and digital) objects being included. In the most granular representation of a workflow, campaigns and segments match 1:1; pre- (and post-)campaign activities are *per se* not considered as part of a workflow campaign
**Domains of elementary operations and their supplementation by method information** Elementary operations → Basic operations being transformation, measurement and transaction of an object Domain 1: Transformation → Generation of (target) objects → For creating objects with new or other properties being suitable for measurement Domain 2: Measurement → Generation of data (specifying an object) → For proofing successful transformation of objects; for enabling data analysis Domain 3: Transaction → Generation of (spatial) structures → For making (physical and digital) objects findable for transformation and measurement Method information (‘methodology’) → Selection of devices and specification of parameters for making processes functional in the context of domains 1–3
**Operation design according to domains 1–3** Operation design → The design of operations in a workflow segment according to the elementary (domain 1–3) operations on an object Factor → Element value used in (statistical) analysis, corresponding to the term ‘variable’ Design element → Generated factor for transformation, measurement and transaction of an object (mostly in an experiment) Parameter → Element value used for describing the setup of devices for operations, corresponding to the term ‘constant’
**Generation and assignment of object identifiers, to operation design with method information, and measurement values** Identifiers (pre-campaign) to objects (containers) → Making objects identifiable Operation design elements with measurement information (pre-campaign) to objects → Making objects distinctive, i.e. characterize the objects Measurement values (on−/post-campaign) to objects → For recording object traits
**Generation and assignment of operation design codes** → (Pre-campaign) generation and assignment of operation design codes to objects

While values of domain 1 to 3 design elements are usually generated by declaration before the corresponding operations, values by measurement are generated during the respective activity. It should be pointed out that aside from *à priori* design being part of workflows as treated here there also exist *à posteriori* design data that might be used as factors in subsequent data analysis. The latter concerns the assignment of measurement data to classification systems. Examples are operational taxonomic units by DNA sequencing onto taxonomic, phylogenetic and functional classifications, operational functional units (OFUs) onto functional classifications, or spatiotemporal coordinates by global navigation satellite system (GNSS) onto elements, i.e. polygons of thematic layers in a GIS analysis context.

The **method information** (MI) accordingly specifies the treatment details during elementary operations to be performed on an object (Figure 2) and provides the **parameters** implied, which represent the **invariables** in the frame of a study. In accordance to the three types of designs, method information also refers to **transformation** parameters of treatments and devices in the context of object treatment during a field and laboratory workflow segment. Transformation may include fragmentation or any other change of properties of a physical object, or structural or even logical properties of a digital object. Method information also includes specifications of **measurement** parameters that are required for gaining evidence of transformation success, i.e. quality and quantity control, but also for generating measurement values for scientific analysis. The **transaction** parameters concerning transfer details of objects are further specified by method information as well. Transaction protocols refer to the transfer of samples into a storage device or to the translocation of objects in general.

For the reason of repeatability of designs, such protocols describing details of the treatment of physical and digital objects are required. Information on processual details are normally provided in laboratory protocols, which are referenced in the methodological chapters of original research articles.

**Operation design elements**, i.e. research study descriptors, concern the selected or declared **factors** in data analyses. In contrast, **method information** with **parameters** describes the details of treatment. Thus, design and methods describe the processual context of an object of workflow segments. Design elements may be based on specific classifications or combinations thereof. The scenarios are potentially infinite in number and mostly refer to the spatiotemporal patterns and hierarchies of experimental setups. They may follow standard terminologies, taxonomies and ontologies and may be supplemented via **semantic enrichment** through resources in internal networks and the internet, normally defined by Uniform Resource Identifiers (URIs).

Aside from the described domains for study design specification and method information, further domains might be recognized like **administrative** research study **details** including legal issues (permits etc.) and **bibliographic data** relevant in the publication context. These domains, however, are not part of research core data and are therefore not considered in the present context.

### Generation of measurement values

**Measurements** on (physical and digital) objects generate **values** according to domain 2 design and corresponding method information. Measurement data are required for hypothesis testing and for quality and quantity control after transformation. A minimum set of measurement data includes measurement values and units and needs to be connected to spatiotemporal coordinates and some more contextual information. For user convenience, it may be organized in an SDMS of its own, separate from domain 1 and 3 data. In such cases, measurement data are linked with transformation and transaction data via shared physical and digital object identifiers. If such a modular solution for scientific data management is applied, it is essential to use these shared identifiers as primary keys in both SDMSs (see example of ‘Software application *DiversityDescriptions* used as SDMS’, further below).

### Object identity and identifiers

Based on the specific study design, **physical** and **digital objects** are generated in each **workflow segment**. They are described by design data and method information (Figure 1; Figure 2; Table 1). For making (physical) objects recognizable in the field, laboratory and for storage, they need to obtain object identity. This is usually insured by labelling their containers with physical **object identifiers**. These labels may include identifiers, which are kept stable, unique and persistent only during the research project lifetime. Increasingly, however, identifiers such as universally unique identifiers, UUIDs (or globally unique identifiers) **(39, 40)** or other persistent identifiers (PIDs) such as URIs or handles like the globally unique and PID for material samples (IGSN), are used already at the start of and along the whole workflow to keep persistence during research project lifetime and beyond, as far as (long-term) data storage, data archiving and data publication are concerned **(40)**. Such types of identifiers are essential for referring to (environmental) objects as well as to generate intermediate (digital) objects and final products, or to label containers thereof, during short- or long-term storage (Figure 2, 3) and are also useful for documenting parent identity relationships (Figure 1). Unique object identifiers are also recommended to be used as primary keys in SDMSs along with design code assignment to objects (see further below). **Universally unique identifiers** as UUIDs of physical or digital objects are explicitly used for making an object distinctive **(41, 30)** and lack semantics. They are essential for data exchange in heterogeneous data networks. They may be regarded as inconvenient for human readability. However, **UUID version 4 group 1 characters** with eight digits with a likeliness of repetition of 1:8^16^ (=1:4 294 967 296) appear sufficient to be used as human-readable ‘minimum-length’ identifiers in a research project context, particularly when additionally linked to the corresponding full-length identifiers in an SDMS.

### Linking workflow segments, operation designs and methods with design codes

For describing workflow segments, e.g. in an SDMS, the relevant design and method information elements are required to be defined and used as descriptors. Required standard data types are numerical, Boolean, categorical, sequence and text (alphanumerical). For creating the preconditions to record data of a given workflow segment, corresponding descriptors and concepts of numerical and text (alphanumeric) data type need to be fixed (relations between design and methods via generated physical object identifier). Data management during environmental studies is organized in two or multiple stages including preparatory work in the **pre-campaign** phase, during field or laboratory campaigns or workflow segments, as well as during subsequent activities of processing and analysing generated data. Each campaign usually comprises one or few workflow segments. In the pre-campaign phase, **primary key object identifiers** have to be assigned to the corresponding descriptors and descriptor states of design elements and method information. The setup of the workflow segment addresses the elementary operation domains of a given object by operation element definition and (sub-)classification.

As described above, it is important that physical representatives of **object identifiers** (preferably UUIDs) are prepared and attached to objects or object containers (Figure 3). Their digital representatives are used as primary keys in an SDMS and assigned to operation designs, method information and measurement values. In addition, design element values, mostly of the transformation design, may be combined to an **operation design code** having the format of **tuples of** (TF, ME and TA) **element values**. They provide information e.g. on details for a given object to be transferred into the respective container during processes of the respective workflow segment. Such code-type information may be required, for instance, during sampling activities in the field and for controlling the setup of (bio-)assays. Therefore, design codes allow for identifying an object’s processual context and for guiding the operator through the respective workflow step or campaign (Figure 3; Tables 2 and 3). Labels assigned to object containers provide the necessary information in a compact form and allow for supporting manual or (semi-)automated processing in the field and laboratory. They may be unique within individual campaigns or workflow segments, but not necessarily within a complete research project or study. Object identifiers and transformation design codes may also be combined into representations as QR codes or barcodes **(30)**.

**Figure 3:**
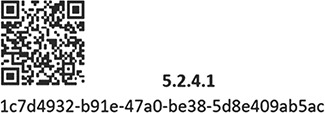
Exemplary label with operation design code (ODC) and identifier (UUID) combined and both represented additionally as QR code. For facilitating the handling during a workflow segment, creation of labels attached on physical object containers (boxes for environmental samples, tubes for laboratory intermediate objects and for storage) needs be achieved beforehand.

**Table 2:** Use case 1 for workflow segments (campaigns). Environmental microbial community barcoding. Domains 1–3: transformation (TF), measurement (ME) and transaction design (TA) elements (E) and method information (MI) elements (E)

**Transformation design (TF)**	**Measurement design (MD)**	**Transaction design (TAD)**	**Method information (MI)**
Workflow segment 1			
E 1: site number/ID	E 1: GPS data	E 1: box ID	E 1 (transformation): sampling protocol
E 2: borehole number/ID		E 2: container ID	E 2 (measurement): GPS (time, space) of borehole at site protocol
E 3: soil horizon type/depth definition			E 3 (transaction): sample into container transfer protocol
E 4: replicate/aliquot number/ID			
Workflow segment 2			
E 1: DNA extraction	E 1: DNA extract concentration and purity	E 1: storage box ID	E 1: nucleic acids extraction protocol
		E 2: storage rack ID	E 2: nucleic acids extract quality and quantity determination protocol
		E 3: microplate ID	E 3: intermediate object transfer into container protocol
		E 4: microplate-internal position coordinate	
Workflow segment 3			
E 1: DNA amplification	E 1: PCR product concentration and product size	E 1: storage box ID	E 1: intermediate object amplification (PCR 1) protocol
		E 2: storage rack ID	E 2: amplicon quality and quantity determination protocol
		E 3: microplate ID	E 3: intermediate object transfer into container protocol
		E 4: microplate-internal position coordinate	
Workflow segment 4			
E 1: PCR amplicon purification	E 1: PCR product concentration and product size	E 1: storage box ID	E 1: amplicon purification (ExoSap digestion) protocol
		E 2: storage rack ID	E 2: purified amplicon quality and quantity determination protocol
		E 3: microplate ID	E 3: intermediate object transfer into container protocol
		E 4: microplate-internal position coordinate	
Workflow segment 5			
E 1: DNA amplification	E 1: PCR product concentration and product size	E 1: storage box ID	E 1: intermediate object amplification (PCR 2) protocol
		E 2: storage rack ID	E 2: amplicon quality and quantity determination protocol
		E 3: microplate ID	E 3: intermediate object transfer into container protocol
		E 4: microplate-internal position coordinate	
Workflow segment 6			
E 1: PCR amplicon purification	E 1: PCR product concentration and product size	E 1: storage box ID	E 1: amplicon purification (magnetic beats) protocol
		E 2: storage rack ID	E 2: purified amplicon quality and quantity determination protocol
		E 3: microplate ID	E 3: intermediate object transfer into container protocol
		E 4: microplate-internal position coordinate	
Workflow segment 7			
E 1: PCR amplicon pooling	E 1: PCR product pool concentration	E 1: storage box ID	E 1: amplicon pooling protocol
		E 2: storage rack ID	E 2: library quality and quantity determination protocol
		E 3: microplate ID	E 3: intermediate object transfer into container protocol
		E 4: microplate-internal position coordinate	
workflow segment 8			
E 1: DNA library storing	E 1: library storage temperature	E 1: storage box ID	E 1: DNA library storage protocol
	E 2: library storage humidity	E 2: storage rack ID	E 2: storage parameters control protocol
	E 3: library storage light	E 3: microplate ID	E 3: product transfer into container protocol
		E 4: microplate-internal position coordinate	

**Table 3:** Use case 2 for workflow segments (campaigns): Fungal isolates, barcoding and phenotypic trait description: Domains 1–3: transformation (TF), measurement (ME) and transaction design (TA) elements (E 1–n) and method information (MI) elements (E 1–3)

**Transformation design (TF)**	**Measurement design (ME)**	**Transaction design (TA)**	**Method information (MI)**
Workflow segment 1			
E 1: site number/ID	E 1: GPS data	E 1: box ID	E 1 (transformation): sampling protocol
E 2: borehole number/ID		E 2: container ID	E 2 (measurement): GPS (time, space) of borehole at site protocol
E 3: soil horizon type/depth definition			E 3 (transaction): sample into container transfer protocol
E 4: replicate/aliquot number/ID			
Workflow segment 2			
E 1: (sub-)culture generation number	E 1: fungal colony growth rate	E 1: microplate storage rack ID	E 1: fungal strain isolation and cultivation protocol
E 2: culture medium type		E 2: microplate ID	E 2: fungal culture growth measurement protocol
E 3: culture medium type variation		E 3: microplate-internal position coordinate	E 3: 1: fungal culture translocation-inoculation measurement protocol
E 4: culture replicate number		E 4: aliquot number/ID	
Workflow segment 3			
E 1: DNA extraction	E 1: DNA extract concentration and purity	E 1: microplate storage rack ID	E 1: nucleic acid extraction protocol
		E 2: microplate ID	E 2: nucleic acid quantity/quality measurement protocol
		E 3: microplate-internal position coordinate	E 3: intermediate object transfer into container
		E 4: aliquot number/ID	
Workflow segment 4			
E 1: DNA amplification	E 1: PCR product concentration and purity	E 1: microplate storage rack ID	E 1: DNA amplification protocol
		E 2: microplate ID	E 2: DNA amplificate quantity/quality measurement protocol
		E 3: microplate-internal position coordinate	E 3: intermediate object transfer into container protocol
		E 4: aliquot number/ID	
Workflow segment 5			
E 1: DNA isolate storage	E 1: PCR product concentration	E 1: room number/ID	E 1: DNA isolates storage protocol
		E 2: freezing device ID	E 2: storage parameters control protocol
		E 3: object/product container ID	E 3: product transfer into container protocol
		E 4: aliquot number/ID	
Workflow segment 6			
E 1: DNA amplicon storage	E 1: PCR product storage temperature	E 1: room number/ID	E 1: DNA amplicon storage protocol
		E 2: freezing device ID	E 2: storage parameters control protocol
		E 3: object/product container ID	E 3: product transfer into container protocol
		E 4: aliquot number/ID	
Workflow segment 7			
E 1: fungal culture staining	E 1: fungal trait 1	E 1: culture storage room number	E 1: culture preparation (staining) for light microscopy protocol
	E 2: fungal trait 2	E 2: culture storage rack ID	E 2: culture measurement protocol with checklist of morphological traits (to be recorded in measurement values database)
	E 3: fungal trait 3	E 3: culture storage shelf number	E 3: product transfer onto slide for LM protocol
	E 4: fungal trait 3 + n	E 4: storage box ID	
Workflow segment 8			
E 1: fungal culture storing	E 1: culture storage temperature	E 1: culture storage room number	E 1: culture storage protocol
	E 2: culture storage humidity	E 2: culture storage rack ID	E 2: storage parameters control protocol
	E 3: culture storage light	E 3: culture storage shelf number	E 3: culture transfer into container protocol
		E 4: storage box ID	

Field and laboratory work implies the generation of intermediate physical (and digital objects) or products by transformation and the generation of measurement values for quality and quantity control or for data analysis during the **campaign or post-campaign** phases. During those campaigns that comprise more than one workflow segment, considerable numbers of physical objects and corresponding data may be generated. Workflows usually end with the storage of physical and digital reference objects in a repository. Resulting digital objects along with applied designs and method parameters, assigned to primary key (physical) object identifiers might be transformed into various technical formats and mapped to standard conceptual schemas and ontologies for subsequent (environmental) scientific analysis, data publication and long-term data deposition. Such thoroughly described digital objects might represent the final and starting stage of the data life cycle **(12, 36)**, see below.

### Two workflow design use case studies: species and community DNA barcoding and cultivation of fungal isolates

The present abstract model describes complete workflows of concatenated segments in environmental research. In the past years, the concept has been tested in field work and laboratory context, e.g. for species and community DNA barcoding, based on an implementation in a generic SDMS. Samples collected in the field may be tissues of organisms (species and community) and various kinds of substrates (e.g. soil, water, air, etc.). Locations of collecting environmental samples have been described by a sequence of hierarchical elements, i.e. a code or number for indicating the individual plots, identifiers or codes of objects on the plots (e.g. soil borehole number, tree individual or organism IDs, etc.), names of target substructures (e.g. soil horizons, plant or animal organs), and optionally, numbers of replicates. For scientific analysis, transformations or treatments of objects are manifold and may include the sectioning of environmental samples or parts thereof, being replicates or aliquots. For an experimental setup, e.g. for determining the growth rates of microbial cultures, a hierarchical design may be chosen as follows: culture generation number, growth medium type, growth medium type variation and growth conditions (e.g. temperature). For species DNA barcoding **(42)** and community DNA barcoding or metabarcoding projects **(43, 44)** or microarray hybridization experiments **(45, 46, 47)**, transformation includes a series of steps. They concern the extraction of nucleic acids, the amplification of DNA or RNA and (for metabarcoding) the pooling of amplicons, resulting in the generation of various intermediate object products (e.g. DNA purified extracts, PCR purified amplicons and DNA libraries) according to protocols, which are usually provided by the manufacturers of laboratory kits. This implies that laboratory work in an ‘omics’ context may be divided into at least three or four workflow segments with corresponding physical intermediate objects, each to be characterized according to design elements for transformation, measurement and transaction as well as to method information.

Use case 1 (environmental microbial community barcoding) provides an overview of an exemplary **omics-driven microbial community barcoding workflow** from sampling in the field to the creation and storage of sets of raw sequence data (Table 2). The operation design of the first workflow segment concerns (a) the position of the sample at a given site (name or ID) by characterizing, (b) object (number or ID) and (c) object part (term). Further, (d) the number of replicates or aliquots may be specified. Transformation in the first workflow segment concerns sample collection itself measurements of the sampling site with geo-coordinates by GNSS (e.g. GPS) or climate parameters by sensors. In subsequent workflow segments, operations on physical objects for instance include nucleic acid extraction, DNA amplification and amplicon pooling plus DNA sequencing, as well as corresponding measurements of the quality, and quantity control of nucleic acid extracts and amplicons by spectrophotometry, and the raw read nucleic acid sequence patterns by a DNA sequencing device. While quality and quantity control measurements during nucleic acid processing are relevant for testing and proving the reliability of data, site parameters (design element values) and sequence patterns (measurement values) represent the raw material for scientific analysis.

Use case 2 (fungal isolates, barcoding and phenotypic trait description) gives a schematic overview of a **use case of isolating fungal strains** from environmental samples, DNA extraction and amplification from fungal cultures for subsequent DNA barcoding to generate fungal marker gene sequence data (Table 3). The transformation design of the first workflow segment largely corresponds with that of Use case 1. It is followed by workflow segments of fungal isolation and cultivation, establishing pure cultures and generating subcultures. Subsequent steps may include laboratory standard procedures of nucleic acid extraction and DNA amplification for DNA sequencing, as well as corresponding measurements for quality and quantity control of nucleic acid extracts and amplicons by spectral photometry and measuring DNA sequences in a sequencing device. In addition, micromorphological and other traits of fungal strains are characterized by applying standard procedures.

For a complete documentation of the work- and dataflow from the field campaign to sequence pattern data in the context of data publishing, it is essential to provide the design data of the various workflow segments as part of generated digital objects. The final objects to be published may include geolocation and other measurements obtained in the field, quality and quantity control data of intermediate objects or products in the laboratory as well as final measurement data like DNA sequence raw and processed data. This entails the need of assigning workflow segment design element values and method information to the corresponding identifiable objects, i.e. samples or intermediate objects. For obtaining the data structure of complete and coherent workflows, successional (digital) objects of a given environmental research workflow have to be concatenated via parent identifiers pointing from the preceding object.

## Software applications for management of operation designs, method information and assigned measurement values

Nearly one decade ago, around 100 companies worldwide had set up and provided **LIMSs** (48). Thus, more than 200 LIMS in the wide sense might exist now, mostly being commercial ones. LIMS applications are devoted to the management of structured data. These software applications and services organize information on laboratory consumables and manage processes as well as information on process parameters and laboratory protocols. Central components mostly include the administrative and organizational domains for object management and for addressing the ways of generating and processing analysis results. The most advanced solutions also provide complete workflow modules and often also comprise modules with traditional functionalities of so-called ‘electronic laboratory notebooks’ for organizing and storing semi-structured information like laboratory protocols. ELNs are widely used in academic research laboratories, being more flexible than most Laboratory Execution Systems, which are applied in industrial laboratories (48).

**SDMSs** in this context (49) (https://en.wikipedia.org/wiki/Laboratory_information_management_system;https://www.limswiki.org/index.php/Scientific_data_management_system) are specifically structured to manage laboratory research data (raw data, analysis result data and documents) including certain functionalities for long-term preservation and archiving. Traditionally, SDMSs are implemented as part of LIMSs. Currently, most of these systems are extended to handle structured research data. Several have interfaces to exchange contextual and bibliographic core data mapped to existing norms and (de facto) standards. Addressed standards are certain ISO norms for geographic and analysis data and certain (community-based) standards as ratified by TDWG, GSC and ASTM committees.

Few **commercial SDMS applications** are committed to manage designs and resulting data like measurement data (see BSSN software, https://www.bssn-software.de/animl-de/, and Limsophy RIMS, https://www.limsophy.com/). Most of the medium- to large-sized environmental research laboratories at governmental agencies and non-university organizations now run mid-term and long-term scientific approaches and have well established in-house system solutions for those studies.

University research groups, however, are often confronted with having temporary employees and forced to focus on short-time research topics. This may sometimes contrast to the growing complexity of data analysis pathways in biology, particularly in meta-omics research. However, there are increasingly options to use interoperable and scalable software, scripts, (sub-)discipline-specific services, web-based subject-specific data management workbenches, analysis platforms and pipelines (e.g. (50), UNITE platform and PlutoF web workbench, both for fungal research (51, 52) and the QIIME 2 pipeline (53) for analysing microbial communities). In addition, platforms providing microservices and virtual research environments (VREs), e.g. with Jupyter and Galaxy components, are spreading (27, 54, 55), as well as for complete bioinformatics workflows (56, 57).

These pipeline softwares, file sharing repositories and data file documentations, however, mostly do not focus on the early stages of operational workflows, their segmentation, and do often not consider design details of the three domains required to ensure the repeatability of studies. Furthermore, the solutions are in general fixed by structure and not flexible enough to scope the variations of hypothesis-driven research study design. Holistic approaches to model a database software solution appropriate to store the study design data and data of all steps of research workflows in a generic form are therefore scarce (58, 59). Summarizing, such software solutions for all-inclusive-data management are recommendable particularly for long-term monitoring projects with an agreed study design.

### Software application *DiversityDescriptions* used as SDMS

**Figure 4:**
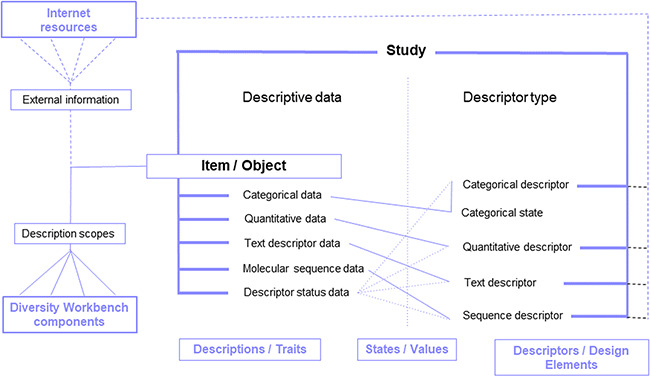
DiversityDescriptions enabling free definition of descriptors and descriptor states for the representation of descriptive data of a study item based on various basic data types and enrichment via ‘description scopes’ by linking Diversity Workbench modules and external web resources.

A software tool for this requested type of **management** of **operation designs**, corresponding data (domains 1–3) and method information is the Diversity Workbench relational SQL-database component *DiversityDescriptions* (DWB-DD) (ver. 4.x, https://diversityworkbench.net/Portal/DiversityDescriptions with manual). The client-server application with rich editing client is part of the Diversity Workbench database framework and is open source and free for download. It is appropriate for modelling and maintaining segmented workflows and creating correspondingly coherent data. Thus, it supports hypothesis-driven designing in environmental research. The setting up of descriptors (concepts) and predefined values (descriptor states) as well as their maintenance is addressed by the DWB-DD ‘descriptor editing interface’. Descriptors may be of categorical, numeric, sequence and text data types. Categorical states (or predefined element values) may correspond with values as, e.g. provided as design code elements (Figure 4). Details on descriptors in general may be continuously added as contextual data, i.e. resource data, using URIs. Designs of all three domains (Figure 1) may exhibit more or less complex hierarchies. The step of establishing a study design in a specific DWB-DD installation should therefore be completed before a study is initiated. Later on, the setting up of descriptions, i.e. the assignment of an object identifier to (predefined) design element values and the measurement values, is achieved via the DWB-DD ‘description editing interface**’**.

Measurement data may be entered into such individually designed SDMS directly via ‘form-editing and grid-editing interfaces’ of the client software or may be imported from XML-formatted documents or CSV-formatted spreadsheet files via data import wizard. Design data of the different domains and corresponding research data (values) may be maintained in separate DD installations building together an SDMS. The connection between corresponding domains of a given object is achieved by applying shared object identifiers (e.g. UUID version 4 group 1 characters) as primary keys or description values. The specification of data elements for identifiers of preceding (parent) objects allows for linking the respective objects to related ones in the workflow. *DiversityDescriptions* allows for sophisticated user rights and access management.

The latest version of the data model of *DiversityDescriptions* has been published in 2016 (60). It largely follows the information model as described earlier (61), is based on an element triple structure and follows the conventions of the TDWG standard for Structured Descriptive Data (SDD: https://www.tdwg.org/standards/sdd/). As part of the Diversity Workbench database framework (62), the *DWB-DD* application is capable of identifying and linking data from other domain-specific modules, following the description scope concept. The linking of DD digital objects with DWB internal and external web resources allows for building a data network with DD objects (Figure 4). Furthermore, *DWB-DD* allows for setting up a project-specific descriptor and value terminology, which in the case of meta-omics research might follow the Meta-omics Data of Collection Objects (MOD-CO) conceptual standard for naming design elements and predefined values for the three design domains (31, 63). Each descriptor and descriptor value might be linked via HTTP URI to external web resources or references respectively and thus allows for linking via HTTP online published laboratory and experiment instructions, protocols and multimedia files (Figure 4).

### Study operation elements mapped to standard conceptual schemas and ontologies, concatenation and coherence of data objects

Study designs depend on scientific questions and the inferred factors (variables) for testing hypotheses. It is rather obvious that design elements and method information, i.e. factors and parameters (invariables), are highly diverse and covered by existing ontologies and conceptual schemas only to some extent. User-driven specifications of design elements for use as factors in subsequent analyses therefore imply that also elements are generated and applied, which are not compliant to existing ontologies with regard to naming and meaning. For data publication, however, it is recommendable that elements of such study-specific proprietary schemas are mapped as far as practicable on elements of community-agreed, service- or domain-specific conceptual standard schemas.

Any existing conceptual schema, allowing the declaration of relations between object identifiers, is principally suitable to explicitly address design elements in a workflow along with method information and measurement data. And indeed, a considerable proportion of proprietary design elements as defined above may be mappable onto generic or domain-specific schemas, and (de facto) standard schemas like ISA Model, AnIML, UnitsML, MIxS, MOD-CO, Ecological Markup Language (EML) and ABCD (64, 65, 66, 67, 68, 31, 69, 70, 71), as well as others, e.g. listed by GFBio under https://gfbio.biowikifarm.net/wiki/Data_exchange_standards,_protocols_and_formats_relevant_for_the_collection_data_domain_within_the_GFBio_network.

Certainly, most of these schemas were designed to publish digital objects from heterogeneous data sources and cover basic functions to make them findable and accessible within and among disciplines using bibliographic data. They often encompass very few schema elements addressing (digital) objects of early scientific workflow stages and workflow segment designs of study operations. Therefore, simple data publication with digital objects compiled in the above-mentioned domain-standards only is mostly not sufficient to guarantee true reusability of data objects and to keep the overall data structure coherent for addressing repeatability of study setup.

There are two steps to realize **concatenation and coherence** of data objects, based on a study-specific proprietary design. As a first step a generic data exchange markup language and format like SDD might be used (72). This DELTA-related TDWG standard schema was ratified in 2005 and allows for organizing descriptions of given digital objects, based on descriptors for various data types, including categorical ones with predefined values. There are, however, few applications that can manage, read and exchange data in this way. One reason for still low acceptance may be that this TDWG standard is considered just to be suitable for ‘data describing a taxon or specimen’. However, SDD can be regarded as a generic standard with XML-based schema, which allows for defining matrix data from all fields of ecological and environmental research.

As a second step to realize coherence, the structured and SDD standardized study operation elements may be **mapped** across multiple conceptual schemas, sometimes with reference to controlled vocabularies, class hierarchies and ontologies like ENVO (73, 74) and MOD-CO (31). During this mapping effort, the first workflow segment has to be considered with priority to keep the coherence respectively concatenation of data objects via child–parent relationships (31).

Functions to **map and convert** descriptors and descriptor values of digital data objects to conceptual schemas and schema elements play an important role as features of a SDMS. The Simple Knowledge Organization (SKOS) provides a common data model and vocabulary for sharing and linking knowledge organization systems like thesauri and classification schemes (75, 76). It is recommended to use parts of this model to optimize **mapping features of the SDMS** used. Thus, descriptor and descriptor state terms can be assigned to alternative schema element terms (i.e. schema concepts) for later use in a Semantic Web data publishing and archiving context. The five SKOS matching categories can be used for labelling semantic relations and the kind of mapping relations (77). This is essential for **matching of concepts**, schema elements and assigned ontology terms to the target schema elements that might not exactly match in every case. Ontology-based data management, **ontology-to-ontology mapping** and a model for conceptual schema transformation from ‘domain schema’ to one or more ‘upper schemas’ exist (78, 79).

### Software application *DiversityDescriptions* used for schema mapping and XML provision of digital objects

*DiversityDescriptions* has a number of features as described above. It might be installed as stand-alone SDMS in a scientific working group and provides mapping options to existing discipline-specific standard schemas and ontologies. Data conversion can be achieved by use of a sophisticated data export wizard. DWB-DD descriptors can be selected and mapped to create a XML schema with compliant XSD document. SKOS categories and certain features of an ODMS are on board.

**Figure 5:**
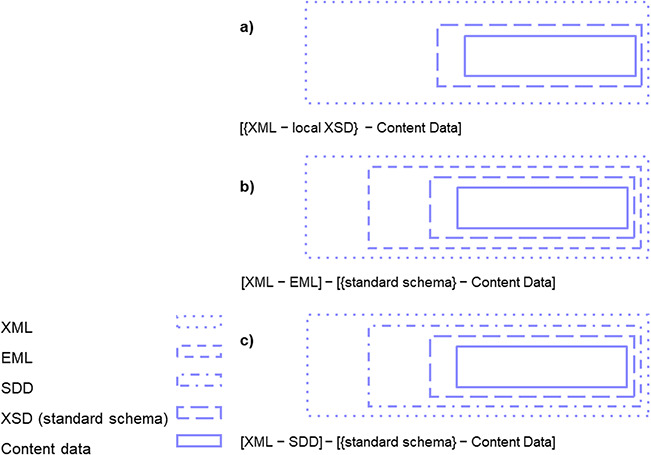
Research data export from DiversityDescriptions provided as XML files with content data with study design-specific vocabulary provided in various formats: (a) as XML (with local XSD), (b) as XML-EML (and core elements mapped to any further domain standard schema) and (c) XML-SDD (and core elements mapped to any further domain standard schema).

Thus, DWB-DD can be used as a flexible tool for the generation of highly structured and coherent digital objects or data products already during the research study, e.g. for serving tools for quality control and analysis. For this purpose, the application is ready to generate any XML file with operation design, method information and measurement values according to a predefined proprietary schema, to validate this schema and to export this file with content data together with the compliant XSD document. This flexible research data export can be done either by using the study design-specific vocabulary for content data or by using elements mapped to any domain standard schema (Figure 5).

Along with other Diversity Workbench components, *DWB-DD* is suitable to integrate internal DWB network information as description scope (Figure 4) and act as core management module in an institutional data repository. An example is the GFBio with data repositories and data centres committed to management, archiving and publication services according to the Open Archival Information System standard (http://www.oais.info/) (36). In this context DWB data pipelines are generating dissemination and archiving information packages. These packages (zip-archives) may include standard schema-formatted SDD-structured XML files for design, method information and measurement values (eventually with elements mapped to MOD-CO) as well as EML-structured files (Figure 5; 16). Bibliographic and administrative (Dublin Core) data elements that are mandatory for GBIF- and GFBio-mediated HTTP URI-resolved data provision may be handled via data repository-specific DWB data publication pipelines (optionally with DOI-assignment). This data handling is not the subject of this article.

## Discussion and conclusion: FAIR++ digital objects

Like in other fields of research, environmental and life sciences research designs are based on scientific questions and hypotheses. Their documentation is essential to ensure the repeatability of studies and potential reproducibility of results. Furthermore, it is indispensable to create and manage highly structured enriched content data already at early stages of the data life cycle. This means, establishment and maintenance of operation data (data structure, administrative, contextual data, ‘metadata’), established before or gathered during campaigns or consecutive workflow segments, with corresponding measurement data, are required to allow for **backtracking** coherent **workflows**.

If taken seriously, proper documentation and linkage of these data to every object generated during a workflow segment is mandatory but has sometimes been neglected in project data management and documentation. For really original scientific studies, designs often need to be unique, i.e. standardized schemas are not applicable. Therefore, study designs in ecology are often considered as too discipline specific for being adequately representable as SDMS pre-set. In consequence, the demand to find adequate conceptual and technical solutions is increasing, to allow for maintaining even specific data with the option of mapping and publishing onto community-agreed schemas later.

Due to the ostensible lack of adequate structures for maintaining coherent data, preliminary strategies to address or circumvent these challenges, like the segregation of data of different context (field work versus laboratory work) or the ‘projection’ of multidimensional structures into two-dimensional matrices entailing a disruption of data relations, are frequently tolerated. In consequence, designs and method information may be disrupted from measurement values or object trait data, which in turn causes impracticality of **backtracking data** published as table or diagram elements. Such lack of traceability is perceived by users of published data, who might have problems with their interpretation, for instance, regarding outliers and the handling of missing data (7). Approaches to restrict publishable research data only to ‘relevant’ or ‘sanitised’ sets of data (6), ignoring that the relevance of published data is principally unforeseeable, is neither considered an adequate solution.

The present abstract model faces this challenge by proposing elementary structures as suitable **backbone** for SDMSs. It includes the assignment of identifiers to objects, the assignment of object identifiers to freely designable design elements and method information, object relations as well as their use to establish **coherence** between consecutive workflow segments. It may be argued that the present abstract model is not necessary, because it is already in parts supported by several existing data management systems and can even be realized by use of spreadsheets. This, however, is not quite correct as even those SDMSs with **broad function scope** often are not flexible enough to provide a frame of elements, which could be defined by investigators themselves as design elements and design element classes (corresponding to schema element definition). In addition, they are not able to face the whole chain or network of relations required for complete coherence in a scientific workflow. This is the same case with “flat structure applications” as spreadsheets, which are rather inconvenient for extended studies and even not stable enough for allowing the management of complex data structures. Wikidata approaches as an option for open data management issues have to be analysed separately.

A SDMS for individual in-house and local, study-intern and institutional management of partly internal and sensible research data is *DiversityDescriptions*. With data publication via internet services, Wikidata is a suitable option to (re-)organize structured digital data objects in a linked-open-data context. The bioschemas.org community project (80, 81) is going to build a semantic layer for retrieval of Life Sciences Websites content through agreed ontologies. Hitherto this project does not have structured FAIR digital data objects from research studies in focus. This might change as soon as it will cooperate with the free knowledge base Wikidata (https://www.wikidata.org) on the retrieval of structured data.

The present abstract model and its prototype software implementation represent an approach, which meets the requirements of specificity and generality. It promotes a type of database application for (a) maintaining and publishing data according to study-specific schemas and workflows, and, due to flexibility, supporting work at a level beyond of what most SDMS solutions allow for, and (b) facilitating the archiving of reusable data by mapping them to domain standard schemas and converting them to archival technical formats. The concept of managing workflow segment design and measurement data together is particularly suitable for keeping overview on ongoing surveys or studies. It reduces the likeliness of wrong assignments of objects to design elements and digital objects by measurement, and, in consequence, contributes to setting up reliable and reproducible data. These are mandatory to fulfil reproducibility requirements and should finally refer to the identifiers of samples and (intermediate) products as deposited in public nature science collections and biobanks (34, 35, 82).

**Figure 6:**
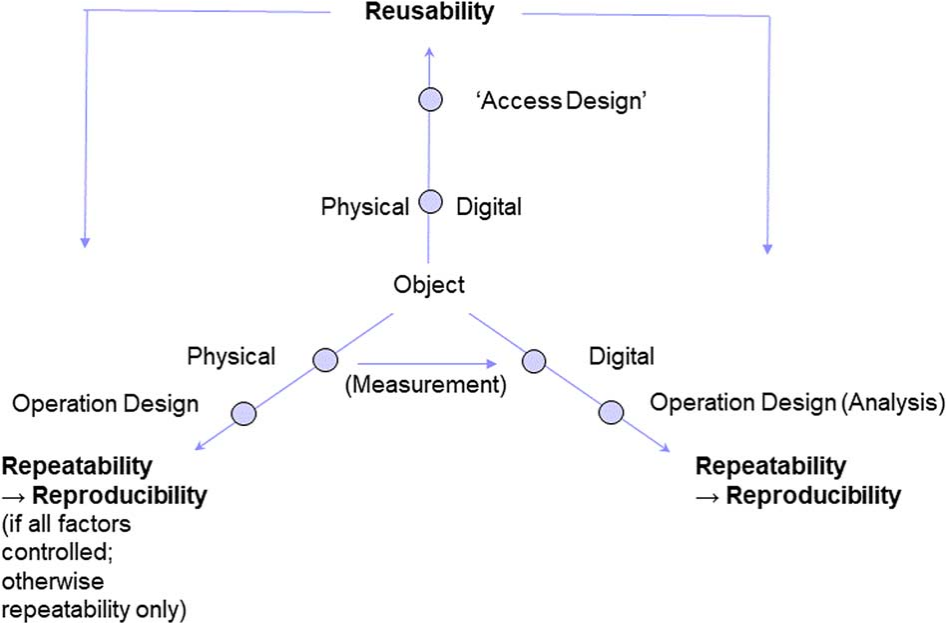
FAIR++: Reusability of physical and digital objects in a research setup is preconditional for the repeatability of operation designs on the respective object type. Analysis of digital objects (according to a certain design), entails the reproducibility of the operation results. Reproducibility of operations on physical objects, however, depends on whether or not environmental parameters are fully controlled. If not, a study setup can be repeated, but results may turn out to be different from the initial ones.

The FAIR guiding principles and recommendations ‘can be applied to a wide range of scholarly outputs’ (13). Confirming this, the European Commission expert group on FAIR data stated that FAIR principles should be applied to any digital objects, which may represent data, software or other research resources and gave a precise definition of the understanding of reusability (83, 84). Mons *et al.* (14) stated that ‘FAIR refers to a set of principles, focused on ensuring that research objects are reusable, and actually will be reused, and so become as valuable as is possible’. Unless ‘reusability’ was widely under discussion as one of the core principles, ‘reproducibility’ has been originally explained with only one example from the publication of so-called ‘non-data research objects’, i.e. to ‘analytical workflows’ (13), and was not at all addressed 1 year later in a subsequent article (14), neither was ‘repeatability’ mentioned.

We therefore recommend to explicitly include ‘repeatability’ (of observation and measurement conditions) and ‘(potential) reproducibility’ (of observation and measurement results) as guiding principles in the FAIR principle context, especially f**or data products or assets**. This implies that the so-called ‘non-data assets’ *sensu* Wilkinson *et al.* like, e.g. scanned documents of lab protocols will come in focus of discussion to be re-structured. According to the proposed abstract model this can be achieved by generating coherent digital data objects with structured and enriched design elements (contextual data). Thus, reusable ‘**FAIR++’** digital objects and data will include detailed design data to guarantee for repeatability and method information to allow for potential reproducibility of research results (Figure 6; 16).

FAIR++ digital data objects of high granularity and coherence as far as freely and openly published on the internet will increase the ‘analytic potential’ of research results (85) and allow for their interpretation also under strict scientific standards.

### Web links and abbreviations

(In alphabetical order; all URLs last accessed at March 15th, 2020)

**AnIML**—Analytical Information Markup Language https://animl.org/

**ASTM**—ASTM Subcommittee E13.15 on Analytical Data https://www.astm.org/COMMIT/SUBCOMMIT/E1315.htm

**ASTM**—ASTM E1578–18, Standard Guide for Laboratory Informatics, ASTM International, West Conshohocken, PA, 2018, https://www.astm.org/

**BIOAWARE**—Life Sciences Data Management Software: https://www.bio-aware.com/

**BSSN software**—https://www.bssn-software.com/

**Capterra**—Capterra software search: https://www.capterra.com/workflow-management-software

**DC**—Dublin Core Metadata Initiative: DCMI Specifications: https://www.dublincore.org/specifications/

**ELN**—Electronic laboratory notebook, in R&D labs, see list of software, e.g. LabCollector.

**EML**—Ecological Metadata Language. https://knb.ecoinformatics.org/tools/eml

**GBIF**—Global Biodiversity Information Facility: https://www.gbif.org/

**GFBio**—German Federation for Biological Data: http://www.gfbio.org

**GFBio Wiki**—Concepts and Standards in the Public Wiki of the German Federation for Biological Data: https://gfbio.biowikifarm.net/wiki/Concepts_and_Standards

**GSC**—Genomic Standards Consortium: https://press3.mcs.anl.gov/gensc/

**INSDC**—International Nucleotide Sequence Database Collaboration: http://www.insdc.org/

**INSPIRE**—INSPIRE Implementation Rules: https://inspire.ec.europa.eu/inspire-implementing-rules/51763

**LabCollector**—https://www.labcollector.com

**IGSN** e.V.—Implementation organization of the IGSN; IGSN is a globally unique and PID for material samples: https://www.igsn.org/

**ISA**—ISA Abstract Model: https://isa-specs.readthedocs.io/en/latest/isamodel.html

**LIMS**—Laboratory information management system—See list of software, e.g. LabCollector.

**LIMS**—Laboratory information management system: https://en.wikipedia.org/wiki/Laboratory_information_management_system

**MendeLIMS**—MendeLIMS: A web-based laboratory information management system for clinical genome sequencing—Scientific Figure on ResearchGate. https://www.researchgate.net/figure/Comparison-among-different-LIMS-systems_tbl1_265093602

**Profeza**—SidSam Profeza Technologies India Private Limited: https://www.profeza.com/

**SDD**—Structured Descriptive Data: https://www.tdwg.org/standards/sdd/

**SKOS**—Simple Knowledge Organization System: http://www.w3.org/2004/02/skos/

**SDMS**—Scientific Data Management System: https://en.wikipedia.org/wiki/Laboratory_informatics

**TDWG**—TDWG group on Biodiversity Information Standards: http://www.tdwg.org/standards

## References

[1] Open Science Collaboration (2015) Estimating the reproducibility of psychological science. Science, 349, 943–945. DOI: 349. 10.1126/science.aac4716. 26315443

[2] Stark P.B. (2018) No reproducibility without preproducibility. Nature, 557, 613. 29795524 10.1038/d41586-018-05256-0

[3] Martin J. (2019) Reproducibility: the search for microbiome standards. BioTechniques, 67, 86–88. 31402678 10.2144/btn-2019-0096

[4] Schnitzer S.A. and Carson W.P. (2016) Would ecology fail the repeatability test? BioScience, 66, 98–99. doi: 10.1093/biosci/biv176.

[5] Fidler F., Wilcox J. (2018) Reproducibility of scientific results The Stanford Encyclopedia of Philosophy (Winter 2018 edition), Zalta E.N. (ed.) https://plato.stanford.edu/archives/win2018/entries/scientific-reproducibility.

[6] Fraser H., Parker T., Nakagawa S. et al. (2018) Questionable research practices in ecology and evolution. PLoS ONE, 13, e0200303. doi: 10.1371/journal.pone.0200303. PMC6047784 30011289

[7] Kang H. (2013) The prevention and handling of the missing data. Korean J. Anesthesiol., 64, 402–406. 23741561 10.4097/kjae.2013.64.5.402 PMC3668100

[8] Hammerling J.A. (2012) A review of medical errors in laboratory diagnostics and where we are today. Lab. Med., 43, 41–44. doi: 10.1309/LM6ER9WJR1IHQAUY.

[9] White E.P., Baldridge E., Brym Z.T. et al. (2013) Nine simple ways to make it easier to (re)use your data. Ideas Ecol. Evol., 6, 1–10. doi: 10.4033/iee.2013.6b.6.f.

[10] Sutter R.D., Wainscott S.B., Boetsch J.R. et al. (2015) Practical guidance for integrating data management into long-term ecological monitoring projects. Wildl. Soc. Bull., 30, 451–463. doi: 10.1002/wsb.548.

[11] Cheah P.Y., Day N.P.J., Parker M. et al. (2017) (2017) Sharing individual-level health research data: experiences, challenges and a research agenda. ABR, 9, 393–400 10.1007/s41649-017-0029-5. 29354190 PMC5746586

[12] Diepenbroek M., Glöckner F., Grobe P., et al. (2014) Towards an integrated biodiversity and ecological research data management and archiving platform: The German Federation for the Curation of Biological Data (GFBio). In: Plödereder, E., Grunske, L., Schneider, E. and Ull, D. (eds). Informatik 2014–*Big Data Komplexität meistern. GI-Edition: Lecture Notes in Informatics (LNI)–Proceedings*, 232, 1711–1724.17.

[13] Wilkinson M.D., Dumontier M., Aalbersberg I.J. et al. (2016) The FAIR guiding principles for scientific data management and stewardship. Sci. Data, 3, 160018. doi: 10.1038/sdata.2016.18. 26978244 PMC4792175

[14] Mons B., Neylon C., Velterop J. et al. (2017) Cloudy, increasingly FAIR; revisiting the FAIR Data guiding principles for the European Open Science Cloud. Inform. Service. Use, 37, 49–56. doi: 10.3233/ISU-170824.

[15] Sansone S.-A., McQuilton P., Rocca-Serra P. et al. (2018) FAIRsharing: working with and for the community to describe and link data standards, repositories and policies. BioRxiv. doi: 10.1101/245183.

[16] Harjes J., Triebel D., Link A. et al. (2019) FAIR data in meta-omics research: using the MOD-CO schema to describe structural and operational elements of workflows from field to publication. Biodivers. Inform. Sci. Standards, 3, e37596. doi: 10.3897/biss.3.37596.

[17] Assante M., Candela L., Castelli D. and Tani A. (2016) Are scientific data repositories coping with research data publishing? Data Sci. J., 15, 1–24. doi: 10.5334/dsj-2016-006.

[18] Waide R.B., Brunt J.W. and Servilla M.S. (2017) Demystifying the landscape of ecological data repositories in the United States. BioScience, 67, 1044–1051. doi: 10.1093/biosci/bix117.

[19] Parsons M.A., Gødoy Ø., LeDrew E. et al. (2011) A conceptual framework for managing very diverse data for complex, interdisciplinary science. J. Inf. Sci., 37, 555–569. doi: 10.1177/0165551511412705.

[20] Leonelli S. (2018) Re-thinking reproducibility as a criterion for research quality. Research in the history of economic thought and methodology: including a symposium on Mary Morgan: curiosity, imagination, and surprise. Res. History Econ. Thought Methodol., 36B, 129–146.

[21] Sandve G.K., Nekrutenko A., Taylor J. and Hovig E. (2013) Ten simple rules for reproducible computational research. PLoS Comput. Biol., 9. doi: 10.1371/journal.pcbi.1003285. PMC3812051 24204232

[22] Renear A.H., Sacchi S. and Wickett K.M. (2010) Definitions of dataset in the scientific and technical literature. Proc. Am. Soc. Inf. Sci. Tech., 47, 1–4. doi: 10.1002/meet.14504701240.

[23] Sacchi S., Wickett K.M., Renear A.H. and Dubin D. (2011) A framework for applying the concept of significant properties to datasets. Proc. Am. Soc. Inf. Sci. Tech., 48, 1–10. doi: 10.1002/meet.2011.14504801148.

[24] Wittenburg P., Strawn G., Mons B. et al. (2019) Digital objects as drivers towards convergence in data infrastructures. EUDAT B2Share. 10.23728/b2share.b605d85809ca45679b110719b6c6cb11.

[25] DONA Foundation (2020) Digital Object Architecture. https://www.dona.net/digitalobject.

[26] Lannom L., Koureas D. and Hardisty A.R. (2020) FAIR data and services in biodiversity science and geoscience. Data Intell., 2, 122–130. doi: 10.1162/dint_a_00034.

[27] Dallmeier-Tiessen S., Khodiyar V. and Murphy F. (2017) Connecting data publication to the research workflow: a preliminary analysis. Int. J. Digit. Curat., 12. doi: 10.2218/ijdc.v12i1.533.

[28] Zeng M.L. (2004) Understanding Metadata. *NISO* National Information Standards Organization, Baltimore, MD http://www.metadataetc.org/metadatabasics/types.htm.

[29] Sansone S.-A., Rocca-Serra P., Field D. et al. (2012) Towards interoperable bioscience data. Nat. Genet., 44, 121–126. doi: 10.1038/ng.1054. 22281772 PMC3428019

[30] Triebel D., Reichert W., Bosert S. et al. (2018) A generic workflow for effective sampling of environmental vouchers with UUID assignment and image processing. Database, 2018, bax096. doi: 10.1093/database/bax096. PMC7206647 29688348

[31] Rambold G., Yilmaz P., Harjes J. et al. (2019) Meta-omics data and collection objects (MOD-CO): a conceptual schema and data model for processing sample data in meta-omics research. Database, 2020, baz002. doi: 10.1093/database/baz002. PMC6354027 30715273

[32] Deutsche Forschungsgemeinschaft – DFG (2015): DFG-Leitlinien zum Umgang mit Forschungsdaten. https://www.dfg.de/foerderung/antrag_gutachter_gremien/antragstellende/nachnutzung_forschungsdaten/index.html with Guidelines on the Handling of Research Data in Biodiversity Research. https://www.dfg.de/download/pdf/foerderung/antragstellung/forschungsdaten/guidelines_biodiversity_research.pdf.

[33] Michener W.K. (2015) Ten simple rules for creating a good data management plan. PLoS Comput. Biol., 11, e1004525. doi: 10.1371/journal.pcbi.1004525. PMC4619636 26492633

[34] Schindel D.E. and Cook J.A. (2018) The next generation of natural history collections. PLoS Biol., 16, e2006125. doi: 10.1371/journal.pbio.2006125. PMC6062129 30011273

[35] Baker M. (2012) Building better biobanks. Nature, 486, 141–146. doi: 10.1038/486141a. 22678297

[36] Grobe P., Gleisberg M., Klasen B. et al. (2019) Long-term reusability of biodiversity and collection data using a national federated data infrastructure. Biodivers. Inform. Sci. Standards, 3, e37414. doi: 10.3897/biss.3.37414.

[37] Maass W., Parsons J., Purao S. et al. (2018) Data-driven meets theory-driven research in the era of big data. Opportunities and challenges for information systems research. J. Assoc. Inf. Syst., 19, art. 1. doi: 10.17705/1jais.00526.

[38] Shah K.R. and Sinha B.K. (2006) Nested Experimental Designs. Encyclopedia of Environmetrics. John Wiley & Sons, Ltd. DOI: 10.1002/9780470057339.

[39] Leach P., Mealling M. and Salz R. (2005) A universally unique identifier (UUID) URN namespace. 'Internet official protocol Standards' (STD 1). Standards Track. https://tools.ietf.org/html/rfc4122.

[40] McMurry J.A., Juty N., Blomberg N. et al. (2017) Identifiers for the 21st century: how to design, provision, and reuse persistent identifiers to maximize utility and impact of life science data. PLoS Biol., 15, e2001414. doi: 10.1371/journal.pbio.2001414. 28662064 PMC5490878

[41] Balkić Z., Šoštarić D. and Horvat G. (2012) GeoHash and UUID identifier for multi-agent systems. Lecture Notes Comput. Sci., 7327, 290–298. doi: 10.1007/978-3-642-30947-2_33.

[42] Liu J., Jiang J., Song S. et al. (2017) Multilocus DNA barcoding—species identification with multilocus data. Sci. Rep., 7, art. no. 16601 https://www.nature.com/articles/s41598-017-16920-2. 10.1038/s41598-017-16920-2 PMC5709489 29192249

[43] Fierer N., Leff J.W., Adams B.J. et al. (2012) Cross-biome metagenomic analyses of soil microbial communities and their functional attributes. Proc. Natl. Acad. Sci. U.S.A., 109, 21390–21395. doi: 10.1073/pnas.1215210110. 23236140 PMC3535587

[44] Peršoh D. (2015) Plant-associated fungal communities in the light of meta-omics. Fungal Diversity, 75, 1–25.

[45] Boch T., Reinwald M., Postina P. et al. (2015) Identification of invasive fungal diseases in immunocompromised patients by combining an *Aspergillus* specific PCR with a multifungal DNA-microarray from primary clinical samples. Mycoses, 58, 735–745. doi: 10.1111/myc.12424. 26497302

[46] Bumgarner R. (2013) DNA microarrays: types, applications and their future. Curr. Protocol. Mol. Biol., 22. doi: 10.1002/0471142727.mb2201s101. PMC4011503 23288464

[47] Sturaro L.L., Gonoi T., Busso-Lopes A.F. et al. (2018) Visible DNA microarray system as an adjunctive molecular test in identification of pathogenic fungi directly from a blood culture bottle. J. Clin. Microbiol., 56. doi: 10.1128/JCM.01908-17. PMC5925724 29514940

[48] Skobelev D.O., Zaytseva T.M., Kozlov A.D. et al. (2011) Laboratory information management systems in the work of the analytic laboratory. Meas. Technique., 53, 1182–1189.

[49] Heyward J.E. II (2009) Selection of a Scientific Data Management System (SDMS) Based on User Requirements. Indiana University-Purdue University Indianapolis 5 pp. https://scholarworks.iupui.edu/handle/1805/2000.

[50] Patrick D., Schloss S.L., Westcott T. et al. (2009) Introducing mothur: open-source, platform-independent, community-supported software for describing and comparing microbial communities. Appl. Environ. Microbiol., 75, 7537–7541. doi: 10.1128/AEM.01541-09. 19801464 PMC2786419

[51] Abarenkov K., Tedersoo L., Nilsson R.H. et al. (2010) PlutoF—a web based workbench for ecological and taxonomic research, with an online implementation for fungal ITS sequences. Evol. Bioinform., 6, 189–196.

[52] Nilsson R.H., Larsson K.-H., Taylor A.F.S. et al. (2018) The UNITE database for molecular identification of fungi: handling dark taxa and parallel taxonomic classifications. Nucleic Acids Res., 47, Issue D1. doi: 10.1093/nar/gky1022. PMC6324048 30371820

[53] Bolyen E., Rideout J.R., Dillon M.R. et al. (2019) Reproducible, interactive, scalable and extensible microbiome data science using QIIME 2. Nat. Biotechnol., 37, 852–857. doi: 10.1038/s41587-019-0209-9. 31341288 PMC7015180

[54] Grüning B.A., Rasche E., Rebolledo-Jaramillo B. et al. (2017) Jupyter and galaxy: easing entry barriers into complex data analyses for biomedical researchers. PLoS Computational Biology, 13, e1005425 10.1371/journal.pcbi.1005425. PMC5444614 28542180

[55] Khoonsari P.E., Moreno P. and Bergmann S. (2019) Interoperable and scalable data analysis with microservices: applications in metabolomics. Bioinformatics, 35, 3752–3760. doi: 10.1093/bioinformatics/btz160. 30851093 PMC6761976

[56] Goble C.A., Bhagat J., Aleksejevs S. et al. (2010) myExperiment: a repository and social network for the sharing of bioinformatics workflows. Nucleic Acids Res., 38 (issue suppl. 2), W677–W682. doi: 10.1093/nar/gkq429. 20501605 PMC2896080

[57] Wolstencroft K., Owen S., Krebs O. et al. (2015) SEEK: a systems biology data and model management platform. BMC Syst. Biol., 9, 33. doi: 10.1186/s12918-015-0174-y. 26160520 PMC4702362

[58] Roth A., Jopp R., Schäfer R. and Kramer G.W. (2006) Automated generation of AnIML documents by analytical instruments. JALA, 11, 247–253.

[59] Schäfer B.A., Poetz D. and Kramer G.W. (2004) Documenting laboratory workflows using the analytical information markup language. JALA, 9, 375–381. doi: 10.1016/j.jala.2004.10.003.

[60] Hagedorn G., Plank A., Link A. et al. (2016) *DiversityDescriptions* data model (ver. 3.0.15, 11 July 2016). https://diversityworkbench.net/Portal/DiversityDescriptionsModel_3.0.15.

[61] Hagedorn G. (2007) Structuring descriptive data of organisms—requirement analysis and data models. (Strukturierung organismischer Beschreibungsdaten—Anforderungsanalyse und Informationsmodelle). Ph.D. Thesis, University of Bayreuth. https://epub.uni-bayreuth.de/632/1/Dissertation_Hagedorn_2007.pdf.

[62] Triebel D., Hagedorn G. and Rambold G. (eds) (1999 onwards) Diversity workbench—a virtual research environment for building and accessing biodiversity and environmental data. http://www.diversityworkbench.net.

[63] Rambold G., Yilmaz P., Harjes J. et al. (2018) MOD-CO schema—a conceptual schema for processing sample data in metaomics research (version 1.0). https://mod-co.net/wiki/MOD-CO_Schema_Reference. 10.1093/database/baz002 PMC6354027 30715273

[64] Sansone S.-A., Rocca-Serra P., Gonzalez-Beltran et al. (2016) ISA model and serialization specifications 1.0. Zenodo. 10.5281/zenodo.163640.

[65] Schäfer B. (2018) Data Exchange in the Laboratory of the Future. A glimpse at AnIML and SiLA Wiley Analytical Science, Hoboken, NJ https://analyticalscience.wiley.com/do/10.1002/gitlab.17270/full/.

[66] Celebi I., Dragoset R.A., Olsen K.J. et al. (2010) Improving interoperability by incorporating UnitsML into markup languages. J. Res. Natl. Inst. Standards Technol., 115, 15–22. doi: 10.6028/jres.115.003. PMC4548526 27134778

[67] Yilmaz P., Kottmann R. and Field D. (2011) Minimum information about a marker gene sequence (MIMARKS) and minimum information about any (x) sequence (MIxS) specifications. Nat. Biotechnol., 29, 415–420. 21552244 10.1038/nbt.1823 PMC3367316

[68] Harjes J., Triebel D., Weibulat T. et al. (2018) Managing and publishing fungal community barcoding data by use of the process-oriented schema MOD-CO and a GFBio data publication pipeline. In: Friedrich-Schiller-Universität Jena. doi: 10.22032/dbt.37811.

[69] Holetschek J., Dröge G., Güntsch A. et al. (2012) The ABCD of rich data access to natural history collections. Plant Biosyst., 146, 771–779.

[70] Fegraus E.H., Andelman S., Jones M.B. and Schildhauer M. (2005) Maximizing the value of ecological data with structured metadata: an introduction to ecological metadata language (EML) and principles for metadata creation. ESA Bull., 86, 158–168.

[71] Fichtmüller D., Berendsohn W., Dröge , et al. (2019) ABCD 3.0 Ready to use Biodivers. Inf. Sci. Standards, 3, e37214 Pensoft Publishers, Sofia, Bulgaria. doi: 10.3897/biss.3.37214.

[72] Hagedorn G., Thiele K., Morris R., Heidorn P.B. (2005) Structured descriptive data (SDD) w3c-xml-schema, version 1.0. Biodiversity information standards (TDWG) http://www.tdwg.org/standards/116

[73] Buttigieg P.L., Morrison N., Smith B. et al. (2013) The environment ontology: contextualising biological and biomedical entities. J. Biomed. Semantics, 4, 43. doi: 10.1186/2041-1480-4-43. 24330602 PMC3904460

[74] Walls R.L., Deck J., Guralnick R. et al. (2014) Semantics in support of biodiversity knowledge discovery: an introduction to the biological collections ontology and related ontologies. PLoS One, 9, e89606. doi: 10.1371/journal.pone.0089606. PMC3940615 24595056

[75] Miles A., Bechhofer S. (2009) SKOS simple knowledge organization system reference, W3C recommendation, world wide web consortium, 18 August 2009. URL: http://www.w3.org/TR/skos-reference/

[76] Baker T., Bechhofer S., Isaac A. et al. (2013) Key choices in the design of simple knowledge organization system (SKOS). J. Web Semant., 20, 35–49. doi: 10.1016/j.websem.2013.05.001.

[77] Sun H., De Roo J., Twagirumukiza M., et al. (2013) Validation rules for assessing and improving SKOS mapping quality. https://arxiv.org/abs/1310.4156.

[78] Lenzerini M. (2011) Ontology-based data management. CIKM, 2011, 5–6.

[79] Calvanese D., Kalayci T.E., Montali M., Santoso A., van der Aalst W. (2018) Conceptual schema transformation in ontology-based data access In: Faron Zucker C., Ghidini C., Napoli A. and Toussaint Y. (ed.) Proc. of the 21st Int. Conf. on Knowledge Engineering and Knowledge Management (EKAW). Lecture Notes in Computer Science, Springer, Basel, Switzerland.

[80] Gray A.J.G., Goble C.A. and Jimenez R., 2017 Bioschemas: from potato salad to protein annotation In International Semantic Web Conference (Posters, Demos & Industry Tracks). https://bioschemas.org.

[81] Michel F. and The Bioschemas Community (2018) Bioschemas & Schema.org: a lightweight semantic layer for life sciences websites. Biodivers. Inf. Sci. Standards, 2, e25836 10.3897/biss.2.25836.

[82] Güntsch A., Hyam R., Hagedorn G. et al. (2017) Actionable, long-term stable and semantic web compatible identifiers for access to biological collection objects. Database, 2017, 1–9. 10.1093/database/bax003 PMC5467547 28365724

[83] RTD (Directorate-General for Research and Innovation) (2018) Turning FAIR Into Reality. Final Report and Action Plan From the European Commission Expert Group on FAIR Data. Publication Office of the European Union, Luxembourg, Luxembourg https://op.europa.eu/en/publication-detail/-/publication/7769a148-f1f6-11e8-9982-01aa75ed71a1/language-en (accessed December 22, 2019).

[84] Lamprecht A.-L., Garcia L., Kuzak M. et al. (2019) Towards FAIR principles for research software. Data Sci., 2019, 1–23. doi: 10.3233/DS-190026.

[85] Palmer C.L., Weber N.M. and Cragin M.H. (2011) The analytic potential of scientific data: understanding re-use value. Proc. Am. Soc. Inf. Sci. Tech., 48, 1–10. doi: 10.1002/meet.2011.14504801174.

